# Importance of Hydrophobic Cavities in Allosteric Regulation of Formylglycinamide Synthetase: Insight from Xenon Trapping and Statistical Coupling Analysis

**DOI:** 10.1371/journal.pone.0077781

**Published:** 2013-11-01

**Authors:** Ajay Singh Tanwar, Venuka Durani Goyal, Deepanshu Choudhary, Santosh Panjikar, Ruchi Anand

**Affiliations:** 1 Department of Chemistry, Indian Institute of Technology Bombay, Mumbai, India; 2 Australian Synchrotron, Clayton, Australia; 3 Department of Biochemistry and Molecular Biology, Monash University, Victoria, Australia; Indian Institute of Science, India

## Abstract

Formylglycinamide ribonucleotide amidotransferase (FGAR-AT) is a 140 kDa bi-functional enzyme involved in a coupled reaction, where the glutaminase active site produces ammonia that is subsequently utilized to convert FGAR to its corresponding amidine in an ATP assisted fashion. The structure of FGAR-AT has been previously determined in an inactive state and the mechanism of activation remains largely unknown. In the current study, hydrophobic cavities were used as markers to identify regions involved in domain movements that facilitate catalytic coupling and subsequent activation of the enzyme. Three internal hydrophobic cavities were located by xenon trapping experiments on FGAR-AT crystals and further, these cavities were perturbed via site-directed mutagenesis. Biophysical characterization of the mutants demonstrated that two of these three voids are crucial for stability and function of the protein, although being ∼20 Å from the active centers. Interestingly, correlation analysis corroborated the experimental findings, and revealed that amino acids lining the functionally important cavities form correlated sets (co-evolving residues) that connect these regions to the amidotransferase active center. It was further proposed that the first cavity is transient and allows for breathing motion to occur and thereby serves as an allosteric hotspot. In contrast, the third cavity which lacks correlated residues was found to be highly plastic and accommodated steric congestion by local adjustment of the structure without affecting either stability or activity.

## Introduction

An efficient strategy employed by nature to sequester unstable intermediates along various biosynthetic pathways is by evolving separate enzymes with coupled reactions that are only active in consort as multiprotein complexes [1], [2]. On the other hand, in some instances analogous systems have emerged that retain these activities together via synthesis of the enzymes as a single polypeptide chain [3], [4]. However, conjoining various domains with multiple activities not only leads to complex folding and unfolding profiles, but also results in creation of interfaces that play a critical role in coordinating function of spatially distant active centers. As a consequence of these complexities caused by domain interactions, there are very few multi-domain proteins for which stability, unfolding mechanism and allosteric regulation have been investigated in detail [5], [6]. An analysis of genomes and present protein sequence databases suggests that 40–60% of proteins exist in multidomain format highlighting the importance of studying such systems [6], [7]. Efforts to understand and mimic such systems have been also pursued for optimizing the production of molecules important from the perspective of industry and medicine [8], [9].

In order to understand multidomain proteins, it is important to understand various aspects of its evolution which allow it to combine various functional units, thereby conferring important properties like stability, catalytic coupling and interdomain communication. Solving the X-ray structure of the protein reveals the hierarchy of the secondary, tertiary and quaternary organization. However, the underlining evolutionary links via which various activities and domains are connected remain elusive. Recently, a new kind of structural classification based on conservation and correlation of amino acids has been described by Ranganathan and coworkers [10]. This method highlighted that proteins retain evolutionary histories, although most of the amino acids evolve independently, there is a small percentage of residues which undergo co-evolution. Statistical coupling analysis (SCA) utilizes this approach and calculates protein sectors that represent a group of spatially coupled residues that co-evolve, and in many instances represent regions important for structure and function of the protein. Several studies performed on small protein systems have been paramount in demonstrating the importance of sectors towards structural and functional aspects, like catalysis and allosteric regulation [10]–[12]. This technique could be highly beneficial for deciphering co-evolution networks in multidomain proteins that have complex architecture and consist of spatially distinct coupled active centers.

FGAR-AT encoded by the *purL* gene is a multidomain bi-functional enzyme that catalyzes the fourth step of the purine biosynthesis pathway. The FGAR-AT protein from *Salmonella typhimurium* (StPurL) has 1295 amino acid residues and is a single chain protein with three major domains [13], [14]. The C-terminal glutaminase domain produces an ammonia molecule that is consumed at the coupled active site present in the formylglycinamidine ribonucleotide (FGAM) synthetase domain where nitrogen is incorporated into an FGAR molecule converting it into FGAM. While the FGAM synthetase domain and glutaminase domain carry out the two enzymatic reactions within this bi-functional enzyme, the function of the N-terminal domain is not clearly understood. The single chain format of this enzyme is found in eukaryotes and Gram-negative bacteria and is often referred to as large PurL (LPurL) whereas in Gram-positive bacteria and archaebacteria FGAR-AT is a complex of three proteins referred by their gene names: PurS, small PurL (SmPurL), and PurQ [15]–[18]. The protein PurS is homodimeric and is structurally homologous to the N-terminal domain of LPurL. SmPurL carries out the FGAM synthetase activity and PurQ carries out the glutaminase activity. Hence, the three main domains of LPurL protein are true evolutionary domains that have homologs existing as independent proteins. Though the X-ray structure of StPurL was determined few years ago [13], many facets of its function like mechanism of interdomain communication, allosteric regulation and path followed by ammonia still remain ambiguous. Most proteins like StPurL that employ an amidotransferase domain do so in consort with a partner synthetase responsible for consuming ammonia [19], [20]. Since uncontrolled release of ammonia is detrimental to cells, these enzymes have developed mechanisms to regulate ammonia production [21], [22]. Glutaminase domains may be inactivated by improper positioning of the active site residues, or by misorientation of the oxyanion hole. Upon sensing of substrate in the adjacent active center, these amidotransferase domains get activated via interdomain signaling.

In the current study the objective was to identify hydrophobic spaces in the protein that are responsible for domain movements that facilitate activation of the enzyme. Xenon trapping experiments were used as focal points to identify hydrophobic cavities. Significance of the hydrophobic voids as well as the flexibility of residue positions within these cavities, were investigated by performing site directed mutagenesis studies followed by biophysical and functional characterization. Evolutionary statistics including SCA was performed on all available PurL and type-I amidotransferases sequences to generate correlation networks consisting of sets of co-evolving residues that have implications towards enzyme function. Putting together all the experimental and SCA findings, a mechanism of allosteric regulation in the PurL protein was proposed. Being a large multidomain protein, PurL is expected to have a complex folding profile. Hence, aspects of mechanism related to unfolding of StPurL were additionally explored.

## Materials and Methods

### Cloning, Expression and Purification

The pET vector based construct of the native PurL protein was obtained from Steven E. Ealick’s laboratory at Cornell University [13]. Point mutants of the enzyme were made by site directed mutagenesis using the strategy employed by the Quikchange (Stratagene) kits. In cases where this strategy failed, overlap and extension PCR followed by cloning using KpnI and BamHI enzymes was employed. For use of this alternate cloning strategy an alternate template with silent mutations was made to remove some restriction enzyme sites. However, two surface mutations R1266S and G1295W that have no effect on the structure, stability and activity of the protein (Figure S1) were unintentionally introduced at this step. Mutations T683W, L1181W and L1181F also have these benign additional mutations. Phusion high fidelity DNA polymerase (Finnzyme) was used for all PCR reactions. Mutant constructs were verified by appropriate sequencing. The clones were transformed into *E.coli* BL21DE3-834 (gold) competent cells and the expression and purification protocol is similar to that observed previously [13]. Briefly, large scale cultures were grown from an isolated colony and the cells were induced at OD_600_ of approximately 0.8 with 0.3 mM isopropyl-β-D-thiogalactopyranoside for 6 hours at 25°C. After growth the cells were pelleted and sonicated in ice cold binding buffer (50 mM HEPES, 500 mM NaCl, 1 mM imidazole, 1 mM glutamine, pH 7.5) and the cell debris removed by centrifugation at 14000 rpm for 45 min. The cleared lysate was applied to a Ni-nitrilotriacetic acid resin (Qiagen), which was pre-equilibrated in binding buffer and subsequently the resin was washed with 50 mM HEPES, 500 mM NaCl, 5 mM imidazole, 1 mM glutamine, pH 7.5 and the His-tagged protein was eluted after extensive washing with 50 mM HEPES, 200 mM NaCl, 100 mM imidazole, 1 mM glutamine, pH 7.5. The eluted protein was further dialyzed into 20 mM HEPES and 100 mM NaCl to remove imidazole and attain low salt conditions for further characterization. Purity was verified by running a 10% polyacrylamide gel followed by coomassie staining. Due to instability and low expression levels of mutants L1181F and T683W, their protein samples could not be made to the same level of purity as those for the wild type protein.

### X-ray Crystallography

**Figure 1:**
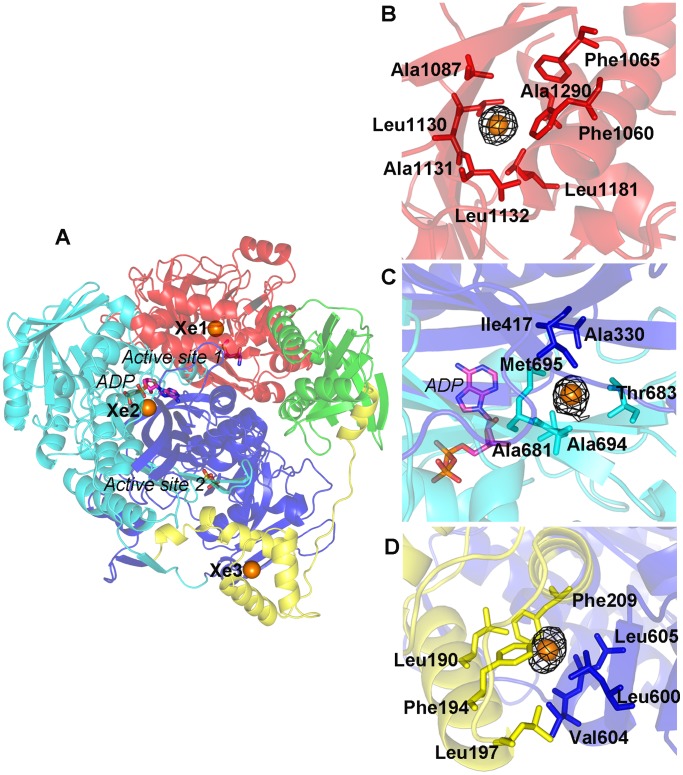
Xenon binding in StPurL. (A) StPurL structure is depicted in cartoon with N-terminal domain in green, linker domain in yellow, glutaminase domain in red, and FGAM synthetase domain with structural sub-domains 1 and 2 shown in blue and cyan colors respectively. The two active sites and the auxillary ADP are shown in sticks and Xenon atoms are depicted as orange spheres. In the side panel (B, C and D), mFo-DFc densitiy maps of the three Xenon atoms at 5.0 σ are shown in black mesh and residues forming the cavities around them are shown in sticks.

Diffraction data for StPurL-Xenon complex was collected at X12 beamline of European Molecular Biology Laboratory (EMBL)-Hamburg. 200 images were collected for each data set. In order to collect this data, crystals of native StPurL were grown by vapor diffusion as described previously [13] and subsequently they were exposed to xenon gas at 40 bar using a pressurization chamber from Hampton Research [23]. The crystals were cryo-cooled immediately after de pressurization and tested for X-ray diffraction at 100 K and the data collected. Multiple data sets were collected with crystals pressurized for different durations ranging from 30 sec to 5 min. The *Auto-Rickshaw* automated crystal structure-determination software [24] was used to confirm at the beamline that the appropriate xenon derivative data had been captured. The crystals pressurized for 1 min diffracted to a resolution of 2.18 Å for the R1263A mutant and 2.65 Å for the native. The larger size of the R1263A mutant crystals enabled collection of higher resolution data perhaps due to larger beam-size at the beamline. Data was collected using a MAR225 detector, 0.5° oscillation, 30 sec exposure time and 150 mm crystal to detector distance at a wavelength of 1.37 Å. Crystals of F209W mutant were also grown by vapor diffusion as described above and diffraction data was collected at BM14 beamline of European Synchrotron Radiation Facility (ESRF), Grenoble. The data for StPurL-Xenon and F209W mutant, like that of the native crystals were in the hexagonal space group P6_5_ with unit cell dimensions a = 146.68 Å, c = 141.22 Å for the xenon complex (PDB ID **4L78**) and a = 146.33 Å, c = 140.84 Å for F209W mutant (PDB ID **4LGY**). The data were indexed, integrated in iMosflm and scaled using SCALA and CCP4 [25] suite of programs. The structures were determined by performing rigid body refinement against the published structure of StPurL (protein data bank (PDB) ID: IT3T) using the program Refmac5 in CCP4i [26] suite. The xenon bound sites were identified by constructing both an anomalous difference Fourier (Figure S2) and mFo-DFc maps (Figure 1). The initial models were subsequently refined by performing rounds of refinement using Refmac5 [27] followed by manual model building using the program Coot [28]. Cavity volumes for the xenon binding sites were calculated using the program Deepview Swiss-PdbViewer [29].

### Circular Dichroism (CD) Spectroscopy

CD spectra were recorded on a Jasco J-815 CD spectrometer using 0.5 mg mL^−1^ protein in phosphate buffer (50 mM sodium phosphate, 100 mM NaCl). Scans were performed at 20°C using 0.1 cm path length quartz cuvettes with 8 sec differential integration time at a scan rate of 50 nm sec^−1^. Thermal denaturation studies were performed by measuring signal at 222 nm while raising the temperature from 20°C to 95°C at 1°C min^−1^ ramp rate.

### Activity Assay

The activity was measured using a previously published protocol [16]. In short, a coupled reaction was used where FGAR was converted into FGAM by PurL, which was then converted to AIR (aminoimidazole ribonucleotide) by PurM protein. The reactions were carried out in 150 µL volume with 50 mM phosphate buffer pH 7.5, 20 mM MgCl_2_, 80 mM KCl, 50 mM L-glutamine, 10 mM ATP, 2 mM FGAR, 5 µl PurL and 5 µl PurM. The reaction was incubated at 37°C for 10 min and quenched by the addition of 25 µl of 20% trichloroacetic acid (pH 1.4). The concentration of AIR was estimated by using a modified Bratton-Marshall assay [16]. The intensity of the pink color was recorded at 500 nm on Spectramax M5 instrument.

### Analysis of Evolutionary Statistics

All the sequences from the SWISS-PROT database that were annotated as PurL were downloaded. Among these, all sequences longer than 1100 residues were classified as LPurL and within these a redundancy cutoff of 90% was applied. These sequences were aligned using ClustalX and constituted the LPurL multiple sequence alignment (MSA). Then, PurL sequences from the SWISS-PROT database that were 500 to 800 residues in length were downloaded and classified as smPurL. A redundancy cutoff of 90% was applied to these too and they were aligned using ClustalX to create the smPurL MSA. Since two PurS units are equivalent to the N-terminal region of LPurL a synthetic PurSS (dimeric PurS) sequence was constructed for creation of the MSA. Subsequently a similar procedure was used to make PurSS and PurQ MSAs. For preparing the PurSSLQ MSA, the 3 MSAs namely PurSS, smPurL, and PurQ were concatenated using PHP language. The LPurL and PurSSLQ MSAs were aligned to each other based on the structural alignment of StPurL (PDB ID: 1T3T) [13] from LPurl MSA and TmPurSSLQ (PDB ID: 3D54) [18] from the PurSSLQ MSA. This structural alignment was carried out using the least square method in Coot [28]. The two MSAs were then carefully appended while maintaining the structure alignment of the two members and also the sequence alignment of the individual MSAs. A sequence identity cutoff of 95% was applied to the resultant MSA to yield the final alignment that was truncated to include only those columns that were occupied in the wild type StPurL sequence corresponding to PDB ID 1T3T. This final MSA consisted of 765 sequences (∼300 LPurLs and ∼400 PurSSLQ multicomplexes) and 1295 positions.

The MSA for type I glutaminases was largely acquired from Pfam. First, the full alignment of the glutamine amidotransferase (GATase) family from Pfam was procured. The glutaminase domain from the earlier PurL MSA was incorporated in the GATase MSA using structure based sequence alignment of PurQ (chain D of PDB ID 3D54) [16] and glutaminase or HisH domain of IGP synthase (PDB ID: 1K9V [30]). HisH was chosen because it had the minimum root mean square deviation (rmsd) value of 2.0 Å with PurQ, among other class-I glutaminases for which structure is known. The final alignment was truncated based on positional occupancy level of 50% and sequences with more than 95% identity were removed and consisted of 8000 diverse sequences. These sequences were analyzed using the Matlab package of SCA [31] version 5.0 developed by Ranganathan *et. al* at University of Texas, Southwestern Medical Centre Dallas and the detailed results used to select sectors are depicted (Figures S3–S6). Perturbation analysis for amino acid level correlations was carried using methods described previously [32].

## Results

### Xenon Trapping Experiments

The native crystals of StPurL protein were exposed to xenon gas and it was noticed that crystals derivatized for 5 min or longer at 40 bar, diffracted worse than 4.0 Å resolution. Moreover, solving the structure of StPurL protein crystals (StPurL-Xenon complex) which were pressurized for 4 min did not show any increase in the number of xenon atoms bound as compared to the crystals derivatized for 1 min therefore, 1 min pressurization of xenon was used for further studies. Data sets of the xenon bound structure of StPurL were collected and refined using both native and a mutant version of StPurL R1263A (Table S1 and Table 1). This mutant was analyzed and was found to be structurally identical to the xenon data set of the native protein (2.65 Å) and diffracted to a higher resolution of 2.18 Å and therefore, used for further analysis. Five xenon atoms were located using mFo-DFc map and confirmed using an anomalous difference fourier map (Figure S2). However, only three out of them were buried in the core (Figure 1A). The other two xenon sites occupied shallow hydrophobic patches on the surface of the protein formed by crystal packing contacts, therefore were deemed functionally unimportant. There were no significant changes in the structure on introduction of xenon and the StPurL-Xenon complex was found to be isomorphous to the native structure (Figure S7).

**Table 1:** Data processing and refinement statistics.

*Data collection*	R1263A- Xenon complex (PDB ID 4L78)	F209W StPurL mutant (PDB ID 4LGY)
Resolution (Å)	2.18	1.48
Multiplicity^a^	6.3 (6.1)	3.9 (3.8)
Completeness (%)^a^	100 (100)	96.5 (94.1)
R_sym_ (%)^a^	14.2 (21.5)	6.7 (24.5)
I/σ^a^	9.1 (6.0)	11.3 (4.7)
Total no. of reflections	565693	1071179
No. of Unique reflections	89982	274143
***Refinement***		
Resolution range (Å)	19.9–2.18	27.5–1.48
no. of reflections total	88494	272850
no. of reflections test set	1067	1114
R_work_/R_free_ (%)	13.9/18.8	14.1/16.6
***No.of atoms***		
total	10897	11924
protein	9897	10142
ligand	30	30
xenon	5	0
ion	73	140
water	892	1612
***rmsd***		
bond lengths (Å)	0.02	0.03
bond angles (°)	2.21	2.72
***Ramachandran plot***		
most favored region (%)	94.9	96.3
additionally allowed region (%)	4.6	3.2
outliers (%)	0.5	0.5

a values for the highest-resolution shell are given in parentheses.

### Analysis of Internal Xenon Binding Sites

The first xenon site (Xe1) was found nested in the hydrophobic barrel characteristic of type I glutaminases in the ammonia production domain that constitutes 24% of the total protein. Overall cavity volume was 63 Å^3^ where the Xe1 atom was buried about 10 Å beneath the surface of the protein and situated approximately 20 Å away from this active site. The walls of the cavity were formed by strands β42, β43, β49 and β50 on one side and helices α30 and α36, on the other side. The xenon moiety was stabilized by the side chains of hydrophobic residues Phe1060, Phe1065, Ala1087, Leu1130, Leu1132 and Leu1181 and the Cα atom of Ala1131and Ala1290 (Figure 1B). A sequence conservation study of the amino acid distribution of the residues in the cavity surrounding the Xe1 site across 295 unique sequences of the LPurL protein showed that these positions were mostly populated by hydrophobic residues. Among these, Leu1181 and Leu1132 were the most conserved, with the position 1181 being occupied by phenylalanine residue 48% of the time and leucine residue 38% of the time. This site was occupied by tryptophan residue in 6% of the sequences.

The second xenon atom (Xe2) was located in the FGAM synthetase domain, trapped at the edge of the hydrophobic barrel formed between the two gene duplicated halves of this domain (Figure 1C). These two halves have 2 fold pseudosymmertry and while domain 1 (A1 and B1 sub-domains) houses the FGAM synthetase active site, domain 2 (A2 and B2 sub-domains) houses an adenosine tri-phosphate (ADP) binding site of unknown function [13]. The xenon binding cavity contains residues from both A1 and A2 sub-domains. Xe2 is stabilized in the hydrophobic cavity formed by residues Ala330 and Ile417 residing on strands β12, β13 and β16 respectively from A1 sub-domain and Ala681, Thr683, Ala694 and Met695 residing on strands β25 and β26 from A2 sub-domain (Figure 1C). Out of these, position 330 was evolutionarily the most conserved and was occupied by an alanine residue in about 95% of the LPurL sequences. Residues Thr683, Ala694 and Met695 were also well conserved and observed in over 80% of the sequences. This was the smallest cavity out of the three internal xenon binding sites; however, since xenon is known to be able to squeeze through small spaces it could accommodate itself in this region [33]. The constrained nature of this site led to lower occupancy of xenon in this site than the others and the anomalous signal for xenon at this site had the lowest intensity out of the three sites.

The third xenon atom (Xe3) was located at the interface of the three helix N-terminal linker domain and the central FGAM synthetase domain. This xenon site was the more solvent accessible as compared to the other two internal xenon atoms, located only about 5 Å under the surface in a 50 Å^3^ cavity. The linker domain is solvent exposed and it forms part of the entrance to the active site cleft where FGAM is produced. This domain is the only region that hangs-off from the structure and breaks the globular symmetry of the protein. Xe3 was sandwiched between helices α7 and α8 of the linker domain and a thirty amino acid linker loop region connecting the B1 and the B2 gene duplicated halves of the FGAM synthetase domain. The side chains of Leu190, Leu197, Leu600, Val604, Leu605, as well as the aromatic rings of Phe194 and Phe209 formed the xenon-binding pocket for Xe3 (Figure 1D). Consensus analysis of the available LPurL genes indicated that this region was largely hydrophobic in nature in all LPurLs.

### Unfolding and Stability of StPurL

**Figure 2:**
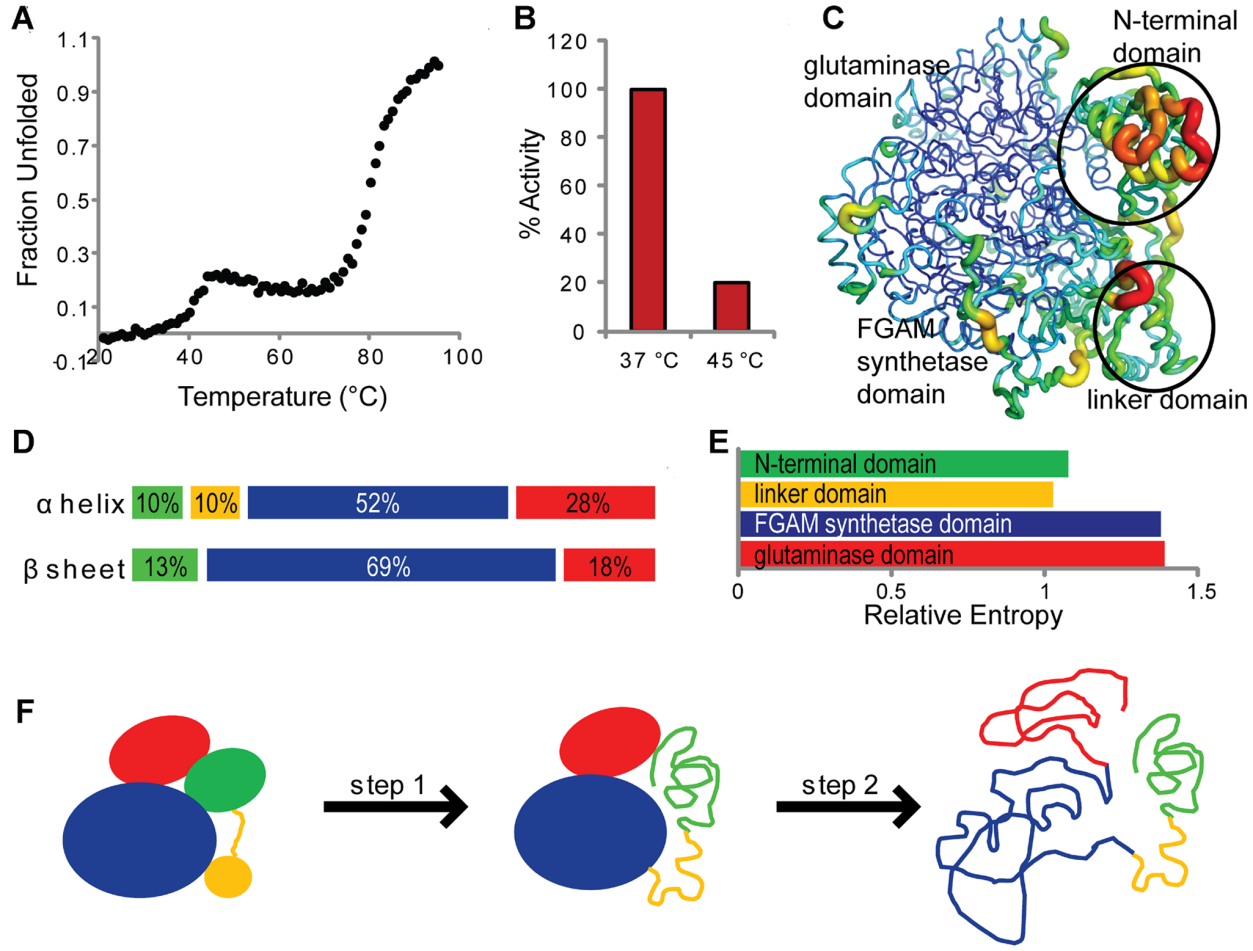
Unfolding and stability of StPurL. (A) Unfolding of StPurL: Thermal denaturation profile of the StPurL protein as observed by measuring ellipticity at 222 nm. (B) Bar graph showing percentage activity (FGAM synthetase) at two different temperatures for StPurL (before and after the first transition) is depicted (C) B-factors: StPurL crystal structure depicted as a function of B-factors. Red, orange and yellow colors represent high B-factors. Blue, cyan and green colors depict low B-factors. (D) Secondary structure: percentage of residues with α helical and β sheet ramachandran angles is pictorially depicted with green color representing N-terminal domain, yellow color depicting linker domain, blue color depicting FGAM synthetase domain and red color depicting glutaminase domain. (E) Conservation: Average relative entropy values representing extent of evolutionary conservation of each domain are plotted. (F) Unfolding mechanism: Proposed unfolding mechanism of StPurL is depicted. The N-terminal domain (green) and linker domain (yellow) unfold in step 1. The FGAM synthetase domain (blue) and glutaminase domain (red) unfold in step 2.

To gauge the stability profile of the StPurL protein thermal denaturation was investigated by following the unfolding of secondary structure elements, more specifically unfolding of α helices, with increasing temperature. The protein appeared to follow a three state unfolding mechanism in two seemingly cooperative steps (Figure 2A). The first unfolding transition at 42°C led to a loss of 15–20% of the secondary structure content of the protein and 80% loss in activity (Figure 2B). The second unfolding transition at 80°C represented unfolding of the bulk of the protein. Domains in a protein may unfold independently or cooperatively and unfolding of multidomain proteins presents few possible scenarios [5]. B-factors of the StPurL crystal structure (PDB code 1T3T) were analyzed to estimate the dynamic nature of various parts of the structure. It was observed that the N-terminal domain had the highest B-factors, followed by the linker domain whereas the glutaminase and the FGAM synthetase domains had lower B-factors (Figure 2C). Moreover, per-residue rmsd calculations between xenon bound and native structures showed a higher degree of variation in the N-terminal region. The above evidence suggests that the N-terminal and the flexible linker domains constituting 15–20% of the secondary structure (Figure 2D), unfold in the first transition at 42°C. Additionally, it has been shown previously that homologs of this protein cannot function efficiently in the absence of PurS protein (analogous to the N-terminal domain) [16] hence, 80% loss of activity upon its unfolding is in accordance with this previous observation. Evolutionary conservation scores calculated using the multiple sequence alignment used for SCA also corroborated this evidence, as the glutaminase and the FGAM synthetase domains exhibited high conservation scores in contrast to the N-terminal domains (Figure 2E). Based on this information, we have proposed a two-step unfolding mechanism for the StPurL protein which is depicted in Figure 2F.

In the proposed unfolding mechanism, the second transition at 80°C is ascribed to the unfolding of both the glutaminase and FGAM synthetase domains. This one step denaturation could indicate one out of the three following scenarios: (1) The domains unfold independently, but only one transition is observed due to their similar stabilities (2) They could be interacting with each other and unfolding as a single cooperative unit with a higher slope as oppose to unfolding of individual domains (3) They could be interacting with each other and not unfold as a single cooperative unit leading to no change in the slope of the transition [5]. To distinguish between these three cases the unfolding profiles of the individual domains is necessary. Hence, based on the present data we cannot determine which out of the three scenarios lead to the second unfolding transition in the PurL denaturation curve. However, since the FGAM synthetase domain and glutaminase domain have an extensive 6280 Å^2^ interface with hydrophobic, polar and ionic interactions and are connected via a compact double helical linker, they are more likely to unfold cooperatively thus favoring the second scenario [13]. Moreover, 80°C is a very high melting temperature for a domain from a mesophilic protein; hence, it is likely that interactions between FGAM synthetase and glutaminase domains have led to higher stability for the multidomain protein than for the individual domains. On the other hand, the N-terminal domain interface is not as extensive and it makes contacts with the FGAM synthetase and the glutaminase domains burying only 1033 Å^2^ and 1919 Å^2^ of the surface area respectively [13].

### Mutagenesis in the Xenon Cavity Region

**Figure 3:**
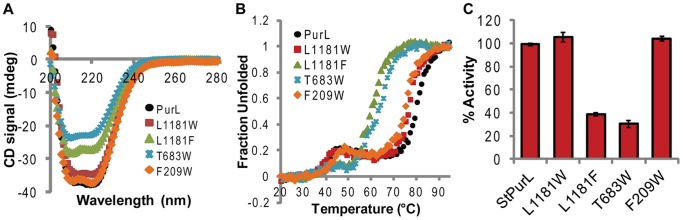
Characterization of StPurLMutants. (A) Secondary structure analysis: Wavelength scan using circular dichroism spectroscopy performed for all variants at the same molar concentration. (B) Stability: Thermal denaturation monitored using CD signal at 222 nm. (C) Activity (FGAM synthetase): Percentage activity of all mutants at 37°C with respect to that of native StPurL protein.

In order to get corroborating evidence for hypotheses about the importance of xenon binding regions mutagenesis studies were performed. These experiments were aimed at perturbing the xenon binding cavities so as to determine their effect on various aspects of protein function and stability. Candidates for site-directed mutagenesis were first screened *in silico* and mutations that showed possible rotamers that could adjust into the structure by filling the cavity without significant clashes with the neighboring residues were chosen. Mutation L1181Y seemed to be one of the most promising case in the *in silico* mutagenesis phase as it did not exhibit significant clashes but filled the cavity. However, the mutant was insoluble and no biophysical data could be collected suggesting that this residue was extremely deleterious to the structural integrity of the protein. As a result of the failure of L1181Y to yield soluble protein, less intrusive mutation L1181F and a bulkier mutation L1181W were subsequently made. Surprisingly the L1181F mutant had lower yield during expression and purification whereas the L1181W was comparable in yield to wild type. The circular dichroism (CD) signal (Figure 3A) also had a lower ellipticity than the wild-type protein for L1181F suggesting lower overall folding of the secondary structure elements for this mutant. Thermal denaturation (Figure 3B) showed that unlike the two step unfolding of the wild-type protein, this mutant had a one step unfolding profile with an unfolding temperature 15°C below the second unfolding transition of the wild-type protein indicating large destabilization. This mutation also caused a 60% loss in activity of the protein (Figure 3C). On the other hand, L1181W had secondary structure content and FGAM synthetase activity at par with the wild-type protein and exhibited almost no destabilization (Figure 3A, C). Thermal denaturation profile was also similar to the wild-type except that the second unfolding transition of the mutant was 5°C below that of the wild-type protein (Figure 3B). This mutagenesis results were intriguing as contrary to general belief, a bulkier residue with obvious clashes was accepted whereas phenylalanine residue with no obvious clash was unacceptable (Figure S8).

Cavity 2 in the FGAM synthetase domain was the smallest and a bulkier mutation T683W was selected as it was expected to fill it substantially. Mutant T683W, like L1181F, also exhibited a lower yield and the CD signal for this mutant (Figure 3A) also showed a lower ellipticity than the wild-type protein indicating disturbance in the secondary structure of the protein. Thermal denaturation profile (Figure 3B) was also similar to a destabilized version of the protein with the unfolding temperature being 12°C below the second unfolding transition of the wild-type protein. This mutation also caused 30% loss in activity of the protein (Figure 3C). The entrance of the Xe2 cavity consisted of two flaps formed by a shorter stretch of residues 685–691 and a long loop spanning residues numbered 643–680. The short loop connecting the strands β25 and β26 of A1 domain was quite surface exposed and the residues within were not conserved in the LPurL family. Unlike the surface exposed nature of the short loop, the forty amino acid long loop runs across the length of the FGAM synthetase domain and connects the A1 domain with the gene duplicated B2 domain and was found to be highly conserved.

Cavity 3 at the interface of the FGAM synthetase and linker domains was fairly large and also quite close to the surface of the protein. Due to this reason, *in silico* analysis showed that it was difficult to fill this cavity as many possible alternate conformations for most residues occupied solvent exposed regions. However, F209W mutation that was expected to block the cavity at the expense of some clashes with the adjacent Leu182 and Glu186 residues was made. Surprisingly, the F209W mutation was very successful and had expression and purification profile at par with the wild-type. Both the secondary structure content and activity of the mutant enzyme was very similar to the wild-type protein (Figure 3A, C). Thermal denaturation profile was also similar to the wild-type except that the second unfolding transition of the mutant was 5°C below that of the wild-type protein (Figure 3B).

**Figure 4:**
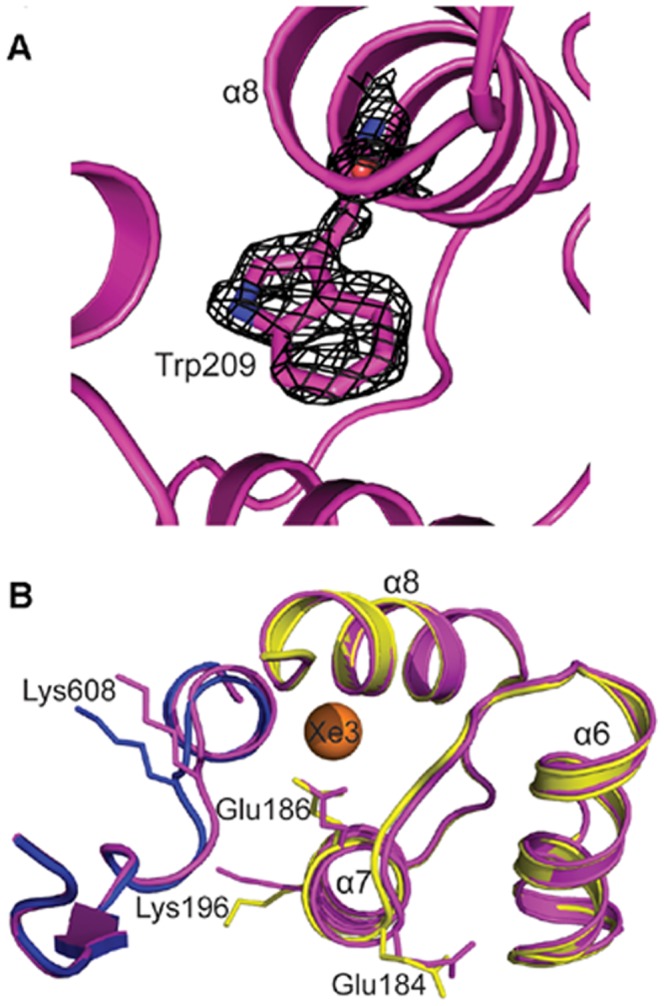
Crystal Structure of F209W mutant. (A) mFo-DFc electron density map for Trp209 at 3.0 σ is shown in black mesh (B) Xe3 cavity region of F209W structure is shown in magenta cartoons and StPurL-Xenon complex is shown color coded with FGAM synthetase domain in blue and linker domain in yellow color. Location of xenon atom is depicted as an orange sphere. Select residues with rmsd of more than 1 Å are shown in sticks.

The crystal structure of this protein was solved by molecular replacement at 1.5 Å resolution. The mFo-DFc map depicted clear density for the tryptophan residue (Figure 4A) that had adjusted into the structure by adopting a similar conformation to that of the phenylalanine residue it replaced. The Cα and Cβ atoms of both the amino acids in both structures superposed exactly. Per residue rmsd of the region around the Xe3 binding cavity was calculated between the F209W mutant and the StPurL-Xenon complex (Figure S9) and it was found that mutagenesis at position 209 resulted in a local readjustment of the structure. To accommodate the larger tryptophan residue, side chains of amino acids in close proximity of the original phenylalanine residue like Glu186 and Leu182 residing on helix α7 readjusted adopting alternate rotamers. The surface exposed lysine residue, Lys608 residing on a loop spanning residues 601–613 which, lines the Xe cavity has the highest overall residue rmsd value of 1.7 Å (Figure 4B). This loop showed maximum perturbation of the backbone in the mutant structure (Figure S9) and is a part of the long linker region (601–631) that connects the B1 and the B2 sub-domains. Other residues in this region with average rmsd over all atoms greater than 1 Å were Glu184, Glu186 and Lys196 (Figure 4B). This result demonstrates that local perturbations in structure can translate into movement of residues in the outer shell of the cavity by propagating adjustments in surface residues and that cavity 3 exhibits structural plasticity.

### Co-evolutionary Statistics

**Figure 5:**
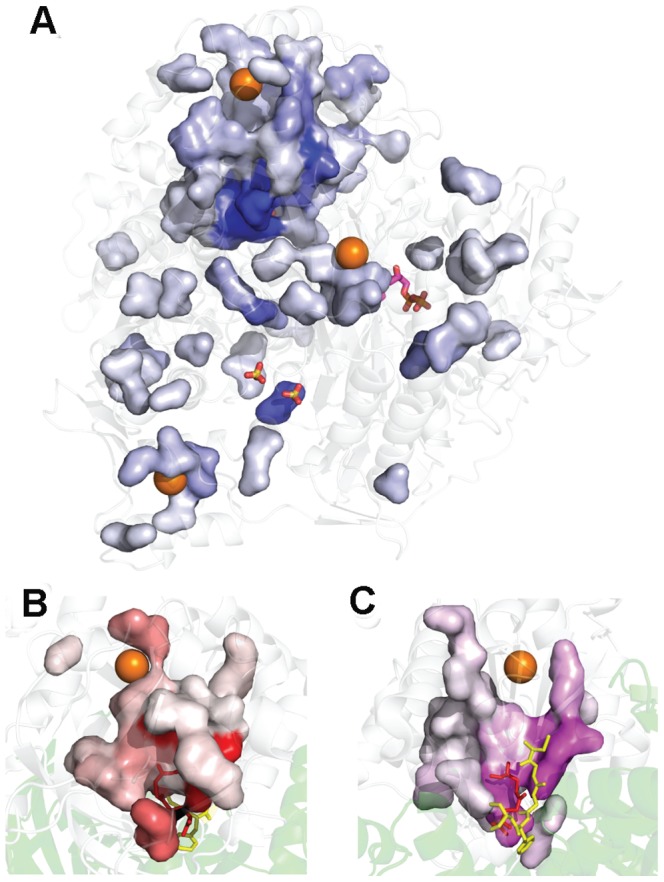
Statistical Coupling Analysis. Sector residues of blue (A), red (B), and magenta (C) are shown in surface representation with darker color in each figure representing higher SCA scores and lighter colors representing lower scores. Xenon atoms are depicted as orange spheres. In (A) locations of the two active sites and auxiliary ADP are shown in sticks. In (B) and (C), residues of the oxyanion hole and the thioester intermediate are depicted in yellow and red sticks respectively.

SCA was performed using programs made available by Ranganathan and coworkers [31]. The PurL MSA was created using both sequence and structural alignment data as described in the material and method section. SCA results yielded four independent sectors (set of co-evolving residues) out of which structural and functional significance could be attributed to two sectors labeled as green (Figure S10) and blue sectors, (Figure 5A). The green sector included multiple residues from each of the xenon binding cavities and this sector included residues from all four domains. While only 48% of the residues of the entire protein were found buried in the crystal structure, over 69% of the residues were buried in the green sector. Conservation scores of the residues were also analyzed using relative entropy metric and it was found that the positions of the green sector were highly conserved (∼90%). High fraction of buried residues and high conservation scores suggest that this sector could represent the hydrophobic core of the protein and may have a role in the stability of the protein.

The second sector termed as the blue sector constituted about 65% of the residues from the glutaminase domain (Figure 5A) and it included catalytically important amino acids belonging to the catalytic triad and the oxyanion hole that connected these regions to the residues lining the Xe1 cavity. L1181, which was subject to various mutagenesis experiments is also a part of this sector. Over 78% residues of these sector residues were buried and 95% of the positions were conserved. The dominant role of the glutaminase domain in the blue sector and also to some extent in the green sector highlighted the importance of this domain and prompted further analysis of the generic type I amidotransferases to which StPurL belongs [20], [34]. Sector analysis was carried out on a large MSA of about 8000 sequences that included amidotransferase domains from various enzymes like carbamoyl-phosphate synthase (CPS) [34], guanosine monophosphate (GMP) synthase [35], HisH subunit of imidazole glycerol phosphate (IGP) synthase [30], cytidine triphosphate synthase [36], 2-amino-2-desoxyisochorismate synthase [37] and anthranilate synthase. SCA of this MSA led to two significant sectors; termed as the red and magenta sectors (Figure 5B, C), 80% of the residues in the red sector and 78% of the residues in the magenta sector overlapped with the blue sector. Both magenta and red sectors included the crucial catalytic triad residues and the red sector in addition encompassed residues from the oxyanion hole, whereas the magenta sector connected the catalytic site with the surface of the protein. In addition, parts of both these sectors lined the central cavity housing the Xe1 site. The magenta allosteric sector touched the cavity on the end closer to the catalytic site (β42, N terminal to the active site cysteine residue. On the other hand, the red sector contained residues lining the bottom of the xenon binding cavity and those in helix α36 which forms the opposite wall of the cavity. Some of the amino acids in the magenta sector were also found to be in the interdomain region formed by the FGAM synthetase and glutaminase domains, in close proximity of the Xe2 engulfing flaps.

## Discussion

StPurL is a 140 kDa protein with multiple domains that catalyzes two concurrent reactions in the purine biosynthetic pathway [13]. As compared to other similar bi-functional proteins like phosphoribosyl pyrophosphate synthase [38] and IGP synthase [30] that also couple ammonia production with other reactions in the nucleic acid synthesis pathways, StPurL is much larger, almost three times the size. This leads to an added layer of complexity in understanding the implications of structure on function. Due to the hydrophobic nature of the xenon atom there have been previous studies where xenon atoms were used to locate hydrophobic spaces. For example, it has been extensively used in the hemoglobin protein system where an apolar channel for oxygen passage and access to the heme-pocket was studied by locating xenon positions using X-ray crystallography [39], [40]. PurL is also involved in channeling of ammonia and therefore it was expected that xenon atoms may line this channel. However, X-ray crystallographic studies revealed that none of the three internal xenon atoms were trapped in either of the proposed ammonia channels within the protein instead they were bound to select hydrophobic spaces within (Figure 1A). Hence, it seems that the channel is inaccessible to external agents in the current conformation. Moreover, xenon trapping results were surprising as only a small number of xenon atoms were found bound to a protein of such a large size. Therefore, it is likely that the compact fold of the protein results in very few surface exposed hydrophobic patches leading to only three internal xenon atoms being structurally trapped. A closer analysis of the three xenon sites along with perturbation of the xenon cavities yielded very intriguing results and provided insights into the mechanism of regulation.

### Regulation of the glutaminase domain

The first xenon site, Xe1, was found bound to the central core of the glutaminase domain, a conserved structural feature found in all type I amidotransferases. Perturbations were made in this cavity to decipher two possibilities; (i) the cavity is an outcome of the fold of this domain and is present to maintain structural integrity and local hydrophobicity (ii) The cavity is dynamic in nature and plays a significant role in regulation of enzyme function. To understand the significance of the Xe1 region we performed mutations at position 1181 that partially filled the cavity. The mutant protein L1181F that posed no steric clash with the surrounding residues (Figure S8) was found to be unstable and exhibited loss of activity. However, contrary to expectations the L1181W whose rotamers had severe clashes with the protein was perfectly accepted and exhibited almost no loss in stability or function (Figure 3). If the first possibility described above was the major reason for the presence of this cavity then filling the core of the protein with analogous hydrophobic residues should not have caused such extreme effects (especially if this substitution did not cause steric clashes) [41]. Therefore, it was proposed that the second scenario was the major contributing factor in this case.

To develop a better understanding into this observation we got to the next level of calculations and invoked correlation effects using evolutionary statistics. SCA provides sectors consisting of correlated set of amino acids that are connected by some property. SCA has been successfully used in the past to ascribe function of sites in the protein which are spatially separated from the active site [11]. SCA performed on the full length PurL indicated that residue L1181 was a part of a sector consisting of correlated residue labeled as the blue sector (Figure 5A). The blue sector connects the hydrophobic cavity, where Xe1 is bound, to the glutaminase active center. This sector also contains both the catalytic triad and oxyanion hole residues that are important for function in type-I glutaminases. The above evidence strongly suggests that the Xe1 site may be important for function because the blue sector connects the active site via a co-evolving network of residues to this cavity. Therefore, it is highly likely that this cavity is involved in cross talk with the active center via the correlated network of residues thereby forming a communication channel. Since, all amidotransferases in the type I family possess the same fold, to decipher if a similar correlation network appears; a separate SCA analysis on a larger data set of 8000 diverse glutaminase sequences was performed. A similar connection of the Xe1 cavity to the active site became apparent in this larger SCA. However, it appeared that the ammonia production center was connected to the Xe1 cavity via two major sectors, red and magenta which were subsets of the blue sector described earlier (only the PurL data) (Figure 5B,C). Moreover, it was discovered that the magenta sector connected the active site to the surface via the wall of the Xe1 cavity. Previous studies have shown that sectors connecting the active site to the surface of the protein could be indicative hotspots for initiation of movements important for allosteric regulation in proteins [12]. Therefore, it is possible that the magenta sector is highlighting regions involved in allosteric activation of PurL. In lieu of the above information it is no longer surprising that perturbation in this region which, in principle is as far as 20 Å away from the catalytic center has a marked effect on activity.

Furthermore, a closer look at the correlation analysis highlighted that positions 1181 (mutagenesis site) and 1286 are strongly correlated in LPurLs. For sequences where 1181 is a leucine residue, position 1286 located opposite it, on β42 strand is almost always a leucine or an isoleucine residue. However, in sequence where position 1181 is occupied by a phenylalanine residue, position 1286 is almost always a methionine residue. Besides, it has been previously shown breaking amino acid correlation networks can yield deleterious effects on certain consensus mutations [42], [43]. Moreover, these co-evolving residues could be a part of a large network, thus posing challenges in designing compensatory mutations due to cascading effects [43]. Since SCA on PurLs indicated that L1181 is part of such an evolutionarily link, it is no longer surprising that stand alone mutations at this site perturb the connectivity, thereby resulting in destabilization. On the other hand, evolutionary statistics reveal that presence of tryptophan residue at position 1181 does not break the correlation analysis even though it occurs with low frequency at this position. Although the X-ray structure for this mutant is not yet available, based on the crystal structure of F209W it can be concluded that L1181W mutation is tolerated by adjustment in structure of the nearby surface exposed loop, via perturbations in the surrounding region without affecting structure, stability and function.

**Figure 6:**
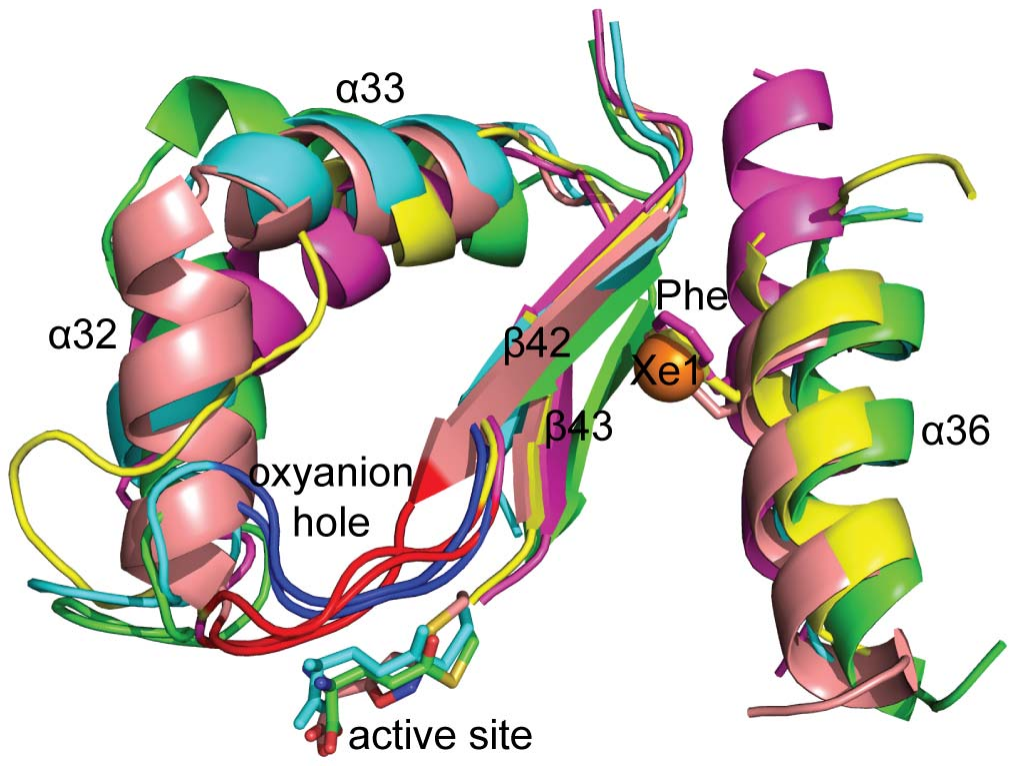
Structural alignment of glutaminase domains. StPurL (PDB file 1T3T) in green color, TmPurQ (PDB file 3D54) in cyan color, IGP synthase (PDB file 1GPW) in salmon color, CPS (PDB file 1C30) in magenta color and GMP synthase (PDB file 1GPM) in yellow color. Secondary structure elements are labeled as per StPurL structure. Active site is demarcated by Cys-glutamine intermediate of StPurL and TmPurQ and inhibitor trapped in IGP synthase shown in sticks. The location of the xenon atom in the StPurL-Xenon complex is shown in an orange sphere and residues blocking the xenon binding pocket in CPS, IGP synthase and GMP synthase are shown in sticks.

In order to propose a model for allosteric regulation via Xe1 cavity, a structural superposition performed on various ammonia producing enzymes CPS (PDB code 1C30) [34], IGP synthase (PDB ID: 1GPW) [30] and PurF (PDB ID: 1GPM) [38]. The structural analyses revealed that this cavity was unique to StPurL, while in other structures this space where xenon was found is filled by none other than a phenylalanine residue (Figure 6). Closer examination revealed that the C-terminal helix of StPurL (α36) adjacent to the Xe1 site was pushed back, and as a consequence did not superimpose well with the helices in the other glutaminase structures (Figure 6) thereby forming the cavity where xenon was bound. A similar scenario was also observed in *thermotoga maritima* (Tm) PurQ where this corresponding helix also superimposed well with StPurL and did not align with the rest of the glutaminases. In addition, it was noted that the loop containing the residues of the oxyanion hole that are important for subsequent hydrolysis of the thioester intermediate, while having a good overlap between CPS, IGP synthase and GMP synthase, had a bad overlap with StPurL and TmPurQ (Figure 6).

Structures of the StPurL protein and the TmPurQ proteins have been reported in an inactive form, where the thioester intermediate is covalently attached to the active site cysteine residue [13], [18]. However, the other representative structures are either in the apo form or with non-covalent inhibitors bound. Therefore, differences in conformation of the helix among these structures (Figure 6) may shed light on some of the movements that take place during allosteric regulation of the protein. Since crystal structure is only a static representation it is possible that the cavity observed in StPurL may provide “breathing space” for these movements to occur and consequently these spaces may be occupied during the course of the reaction. Based on the above evidence we propose that upon activation, a forward motion of helix α36 occurs which, perturbs the beta strand, β42, forming the opposite wall of the xenon binding cavity. This strand is directly connected to the oxyanion hole residues and a perturbation in this region initiates the correct positioning of the active site via restructuring of the oxyanion hole. Hence, we suggest that this cavity is transient and in the alternate conformations of the protein this cavity may be occupied and this space cannot be arbitrarily filled. Furthermore, our results show that only residues that do not hinder with enzyme motion are allowed and correlation analysis can be employed as a potential tool to predict acceptable residues.

### Interdomain Communication in StPurL

**Figure 7:**
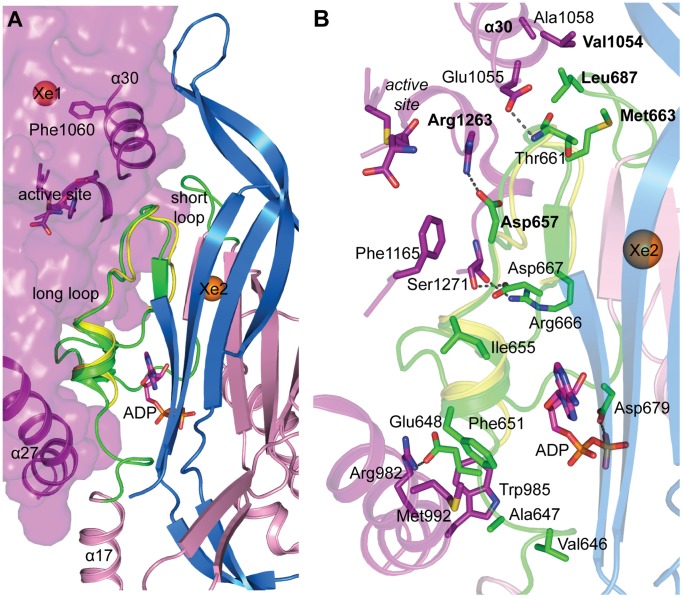
Loop regions around cavity 2. (A) Glutaminase domain is shown in surface representation in purple color with active site residues, helix α27 and α30 highlighted in cartoon and stick representations. A2 domain of the FGAM synthetase is shown in pink cartoons with the long and short loops in green color. The structurally conserved long loop region of TmPurL is shown in yellow cartoon. ADP is shown in magenta sticks. The β-sheet regions of A1 domain involved in making the β-barrel core of the FGAM synthetase domain are shown in marine blue cartoon representation. Locations of the xenon atoms trapped in the structure are depicted as orange spheres. (B) View of the interface between the FGAM synthetase loop regions and the glutaminase domain showing various hydrogen bonding and van der waals interactions.

As mentioned earlier, the Xe2 atom was trapped in a small cavity at the edge of the central β-barrel of the FGAM synthetase domain. The strands constituting the wall of the cavity were connected to two loops, a short and a long one that form a flap. Analysis of the long loop shows that it is buried into the protein and forms part of the interface between glutaminase and the FGAM synthetase domains via a conserved network of interactions (Figure 7A). A comparison of the available crystal structures of TmPurSSQL (PDB ID 3D54) with StPurL further revealed that TmPurL has retained a majority of this long interdomain loop region and that it shows good structural superposition with that of StPurL (Figure 7). The significance of this loop was additionally highlighted, more so because of the thermophilic nature of TmPurL several loops have been deleted in this protein to attain structural stability at higher temperatures, however, this interdomain loop is retained (Figure 7B). Furthermore, it was noticed that the N-terminal region of the long loop is in close contact with the auxiliary ADP binding site and residue Asp679 lying on this loop was in close contact of the second phosphate of the ADP.

A closer look at the structure reveals that the interface formed by the two FGAM synthetase loops with the glutaminase domain is located at the exit path for ammonia. Arg1263 present on top of the glutaminase active site hydrogen bonds to a crystallographic water molecule located at the release point of ammonia, and also with residue Asp657 on this loop (Figure 7B). In addition, both these residues Arg1263 and Asp657 are highly conserved in PurL proteins. Hence, it appears that this loop is indeed a bridge between the two domains and may also be involved in ammonia regulation. Additionally, correlation analysis of this interdomain region also demonstrates that the residues at the interface are highly interrelated. For example, it was found that the amino acids present at this interface, like Met663 residing on the long loop, Val1054 on glutaminase helix α30 and Leu687 on the short loop are part of a correlation network (Figure 7B). The amino acids in these positions are almost always hydrophobic and exhibit a size correspondence; for instance, in sequences where Val1054 is a larger tyrosine residue, Met663 prefers to be a smaller serine or a glycine residue, while Leu687 is almost always replaced by a smaller valine amino acid.

An *in silico* mutation of T683W to block the Xe2 cavity showed that in order to assimilate this mutation, it is likely that the long loop flap of the xenon cavity changes conformation. In light of the importance of this loop region it is not surprising to note that biophysical characterization of T683W showed destabilization of the enzyme and this mutant exhibited loss of FGAM synthetase activity. Therefore, this suggests that non native perturbations near this loop region may lead to improper transfer of signals between the various domains of the protein and may even interfere with auxiliary ADP binding.

### Importance of Hydrophobic Cavities

Out of the three internal hydrophobic spaces marked by xenon atoms, the region around the third xenon site was found to be most malleable and tolerant to mutagenesis. This site was more surface exposed and although mutation of F209W resulted in perturbation of the local environment, the changes were tolerated as is evident from the crystal structure (Figure 4A, B). More than ten residues needed varying degree of adjustment to satisfy a small change in amino acid sequence hence, even a marginal change in size of a single amino acid results in several movements. On the other hand, it can also be concluded from these observations that proteins can adapt to minor changes in structure as long as these changes are transmitted to the surface of the protein and are not involved in consorted movements involved in activation of the protein. Mutations in the hydrophobic voids can have direct implications towards core packing of the protein. This particular mutant of StPurL demonstrated that maintaining hydrophobicity of the local core was sufficient, implying that the core is not a rigid entity and has the ability to breathe and assimilate small perturbation by compensatory motions. This observation is in accordance with the extensive mutation studies carried out in T4 lysozyme by Matthews and coworkers [41] that refute a strict “jigsaw puzzle” packing model and highlight the contribution of the “oil droplet” model of core packing [44], [45].

However, additional mutagenesis studies on the other hydrophobic regions of the protein (like Xe1 and Xe2 cavities), which may be potential allosteric hotspots or have involvement in interdomain communication, showed that these regions of the core are extremely sensitive to structural perturbations. Even seemingly benign change like L1181F, far away from the active centers, can perturb the fold and lead to instability and loss of activity. Positions in these regions cannot be arbitrarily manipulated and are optimized as a set of co-evolving residues. The large difference in stability and solubility of mutations to tyrosine, phenylalanine and tryptophan residues at position 1181 can be explained via a “jigsaw puzzle” model as opposed to “oil droplet” model for hydrophobic core packing. Therefore, it can be inferred that none of the two models are strictly followed by the protein and depending on the overall positioning and sensitivity of a local hydrophobic core region one of these models may be more suitable.

### Conclusion

In this study, we have highlighted the importance of the certain hydrophobic cavities present in the StPurL protein. Xenon trapping was used as a tool to identify these cavities and subsequently these cavities were perturbed via mutations. A detailed analysis of the mutant forms of the protein, using experiments in conjunction with evolutionary statistics along with an in depth inspection of the structure of the protein revealed that the protein possess certain hydrophobic spaces that allow for breathing motion to occur. The residues lining these void spaces form a correlation network that connects them to the active centers thereby forming a communication channel. The results also revealed that hydrophobic spaces in the protein that are not a part of a contiguous network of coupled residues connecting them to the active center, most likely do not play an important role towards function. In contrast to the functionally relevant hydrophobic spaces in these decoupled regions the protein exhibits plasticity and can tolerate structural perturbations even those that directly pose steric clash with the surrounding neighbors. Therefore, correlation analysis along with experiment can be an important approach to identify regions in the protein that play significant role towards various aspects of function like allosteric regulation which, are otherwise difficult to decipher by other means.

### Accession Numbers

Coordinates and structure factors have been deposited in the Protein Data Bank with accession numbers 4L78 for R1263A StPurL Xe Complex and 4LGY for F209W StPurL. The accession numbers for native StPurL-Xenon complex is 4MGH, data included in supplementary material.

## Supporting Information

Figure S1 Characterization of the PurL* construct containing the two unintentionally obtained mutations R1266S and G1295W.
(PDF)

Figure S2 **Anomalous difference Fourier map density of Xe atoms is shown at 5.0 σ for the first four sites and at 3.0 σ for Xe5.**
(PDF)

Figure S3 **Histograms of eigenvalues for the actual alignment.**
(PDF)

Figure S4 **Positions projected along the top four eigenvectors subsequent to independent component analysis step in Statistical Coupling Analysis of PurL protein family.**
(PDF)

Figure S5 **Histograms of eigenvalues for the actual alignment.**
(PDF)

Figure S6 **Positions projected along the top four eigenvectors subsequent to independent component analysis step in Statistical Coupling Analysis of type I glutaminases.**
(PDF)

Figure S7 **Per residue RMSD between crystal structures of native StPurL and R1263A-Xenon complex.**
(PDF)

Figure S8 **Environment of the phenylalanine and tryptophan residues in the Xe1 cavity.**
(PDF)

Figure S9 **Per residue RMSD between crystal structures of native StPurL and StPurL-F209W mutant.**
(PDF)

Figure S10 **Green sector shown in surface representation with the xenon atoms highlighted.**
(PDF)

Table S1 **Data processing and refinement statistics of StPurL-Xenon complex.**
(PDF)
