# Supplementary material for: Importance of Hydrophobic Cavities in Allosteric Regulation of Formylglycinamide Synthetase: Insight from Xenon Trapping and Statistical Coupling Analysis
Source: PLoS One. 2013 Nov 1;8(11):e77781. doi: 10.1371/journal.pone.0077781 (PMC3815217; doi:10.1371/journal.pone.0077781)
Supplement: Figure S1 — Characterization of the PurL* construct containing the two unintentionally obtained mutations R1266S and G1295W. (PDF) [file pone.0077781.s001.pdf]

Figure S1

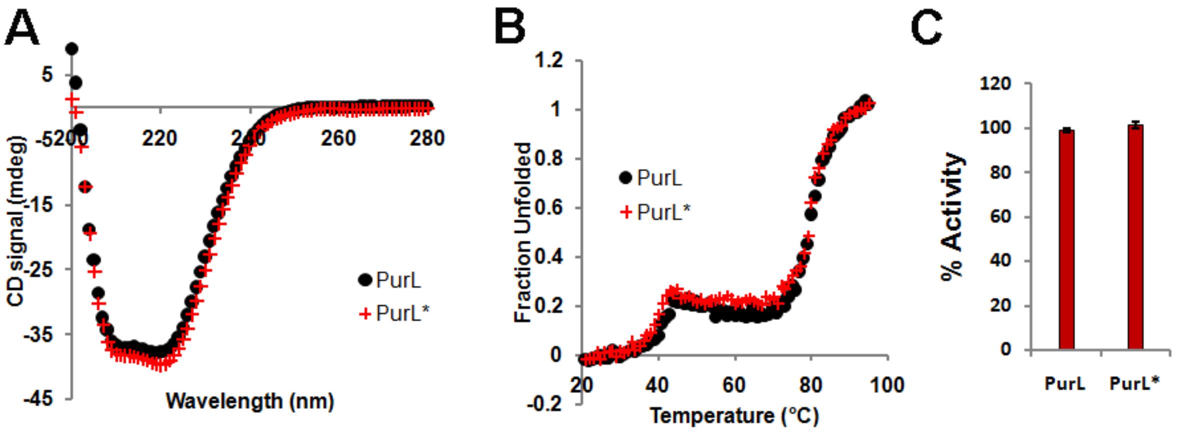

Figure S1: Characterization of the PurL* construct containing the two unintentionally obtained mutations R1266S and G1295W (A) circular dichroism (CD) wavelength scan (B) thermal denaturation profile (C) activity assay. The red cross is data for the surface mutant (PurL*) and black dot indicates data for the native.
