# Supplementary material for: Importance of Hydrophobic Cavities in Allosteric Regulation of Formylglycinamide Synthetase: Insight from Xenon Trapping and Statistical Coupling Analysis
Source: PLoS One. 2013 Nov 1;8(11):e77781. doi: 10.1371/journal.pone.0077781 (PMC3815217; doi:10.1371/journal.pone.0077781)
Supplement: Figure S2 — Anomalous difference Fourier map density of Xe atoms is shown at 5.0 σ for the first four sites and at 3.0 σ for Xe5. (PDF) [file pone.0077781.s002.pdf]

Figure S2

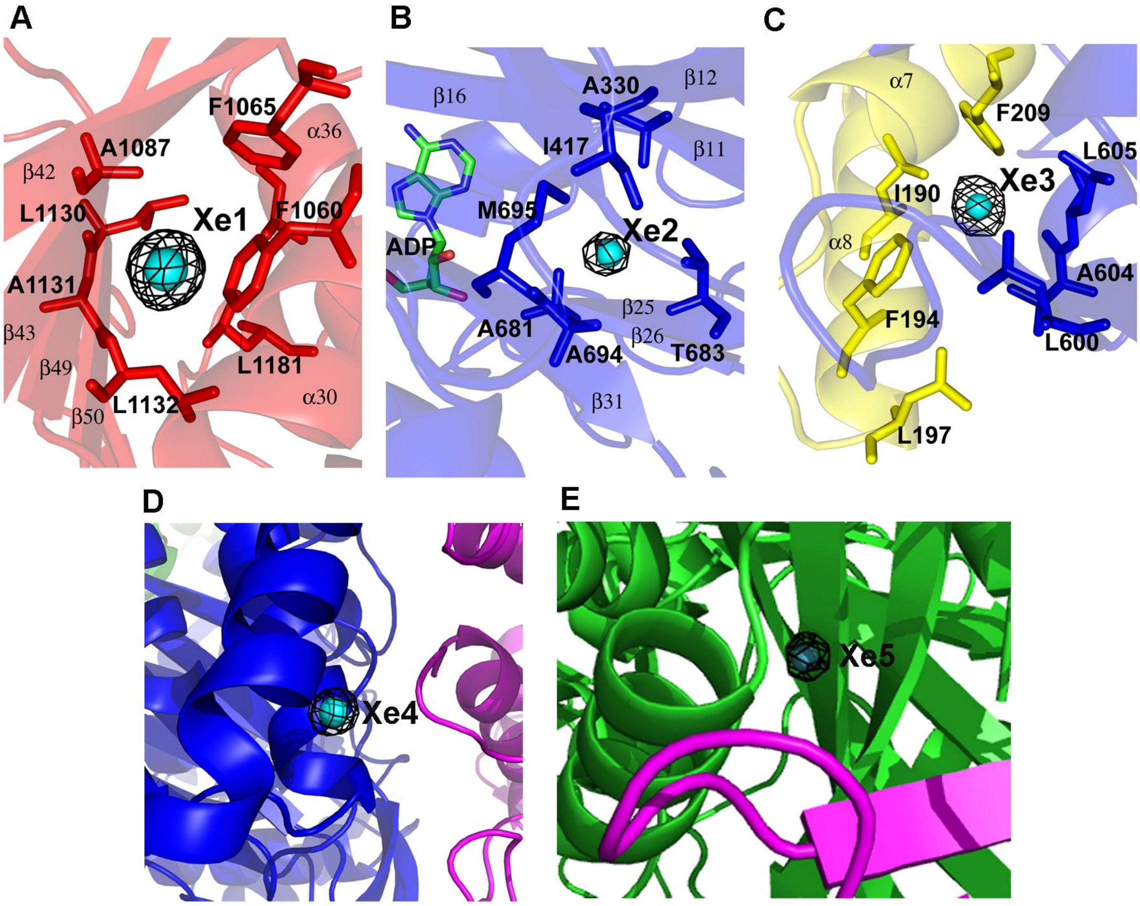

Figure S2: Anomalous difference fourier map density of xenon atoms is shown at 5.0σ for the first four sites and at 3.0σ for Xe5. Internal hydrophobic xenon binding cavities for Xe1 in the glutaminase domain (A), Xe2 in FGAM synthetase domain (B), and Xe3 at the interface of FGAM synthetase and linker domains (C) are shown with cavity forming residues in sticks. The surface sites for xenon binding are shown in cartoon representation with the symmetry related molecule in magenta forming crystal contacts with it. Xe4 is located at the surface of FGAM synthetase domain (D) and Xe5 in the N-terminal domain (E).
