# Supplementary material for: Importance of Hydrophobic Cavities in Allosteric Regulation of Formylglycinamide Synthetase: Insight from Xenon Trapping and Statistical Coupling Analysis
Source: PLoS One. 2013 Nov 1;8(11):e77781. doi: 10.1371/journal.pone.0077781 (PMC3815217; doi:10.1371/journal.pone.0077781)
Supplement: Figure S3 — Histograms of eigenvalues for the actual alignment. (PDF) [file pone.0077781.s003.pdf]

Figure S3

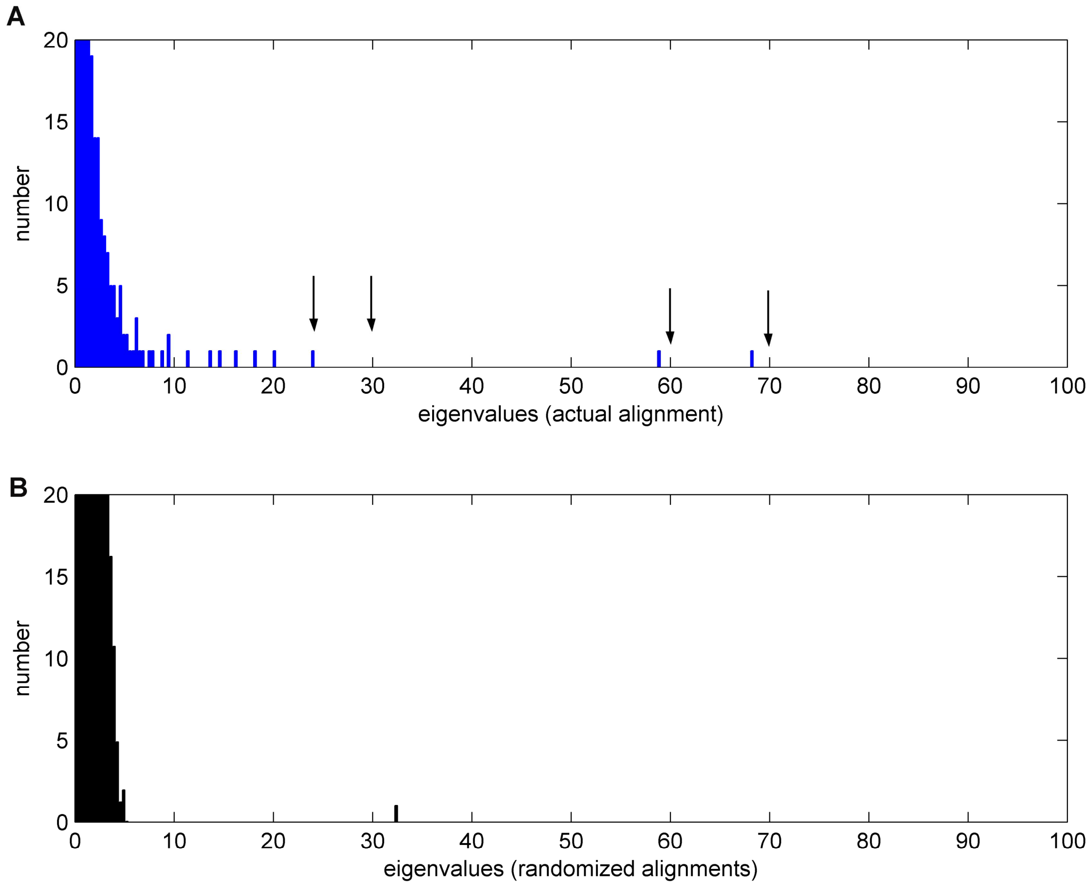

Figure S3: Histograms of eigenvalues for the actual alignment (A) and randomized alignment (B) of the PurL database are shown. Several eigenvalues may be considered statistically significant, however the top four values pointed out in (A) were used.
