# Supplementary material for: Importance of Hydrophobic Cavities in Allosteric Regulation of Formylglycinamide Synthetase: Insight from Xenon Trapping and Statistical Coupling Analysis
Source: PLoS One. 2013 Nov 1;8(11):e77781. doi: 10.1371/journal.pone.0077781 (PMC3815217; doi:10.1371/journal.pone.0077781)
Supplement: Figure S4 — Positions projected along the top four eigenvectors subsequent to independent component analysis step in Statistical Coupling Analysis of PurL protein family. (PDF) [file pone.0077781.s004.pdf]

Figure S4

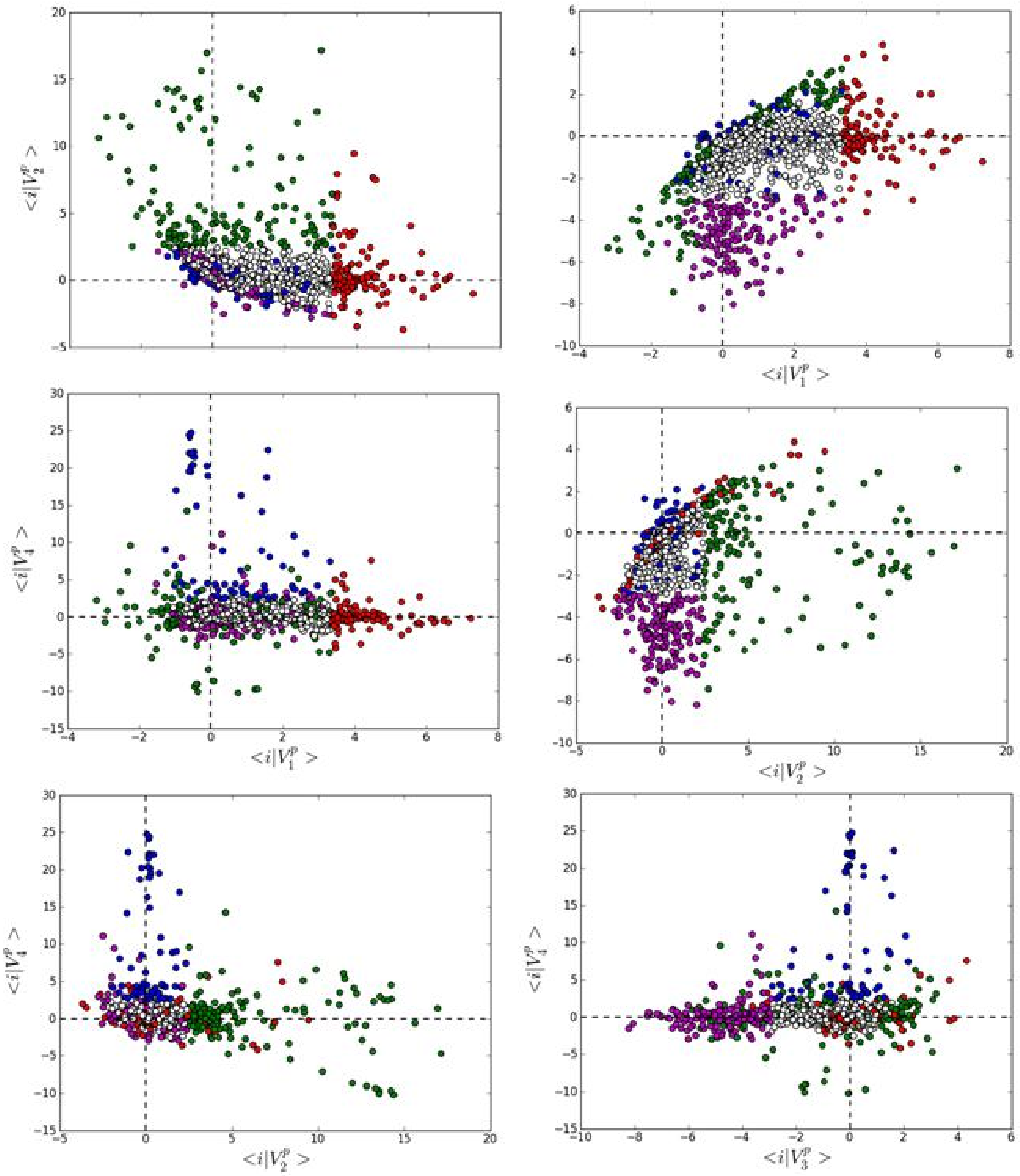

Figure S4: Positions projected along the top four eigenvectors subsequent to independent component analysis step in statistical coupling analysis of PurL protein family. The green dots resolving along eigenvector 2 correspond to the green sector and the blue dots resolving along eigenvector 4 correspond to the blue sector. No functional significance could be attributed to the red and magenta sectors resolving along eigenvectors 1 and 3.
