# Supplementary material for: Importance of Hydrophobic Cavities in Allosteric Regulation of Formylglycinamide Synthetase: Insight from Xenon Trapping and Statistical Coupling Analysis
Source: PLoS One. 2013 Nov 1;8(11):e77781. doi: 10.1371/journal.pone.0077781 (PMC3815217; doi:10.1371/journal.pone.0077781)
Supplement: Figure S5 — Histograms of eigenvalues for the actual alignment. (PDF) [file pone.0077781.s005.pdf]

Figure S5

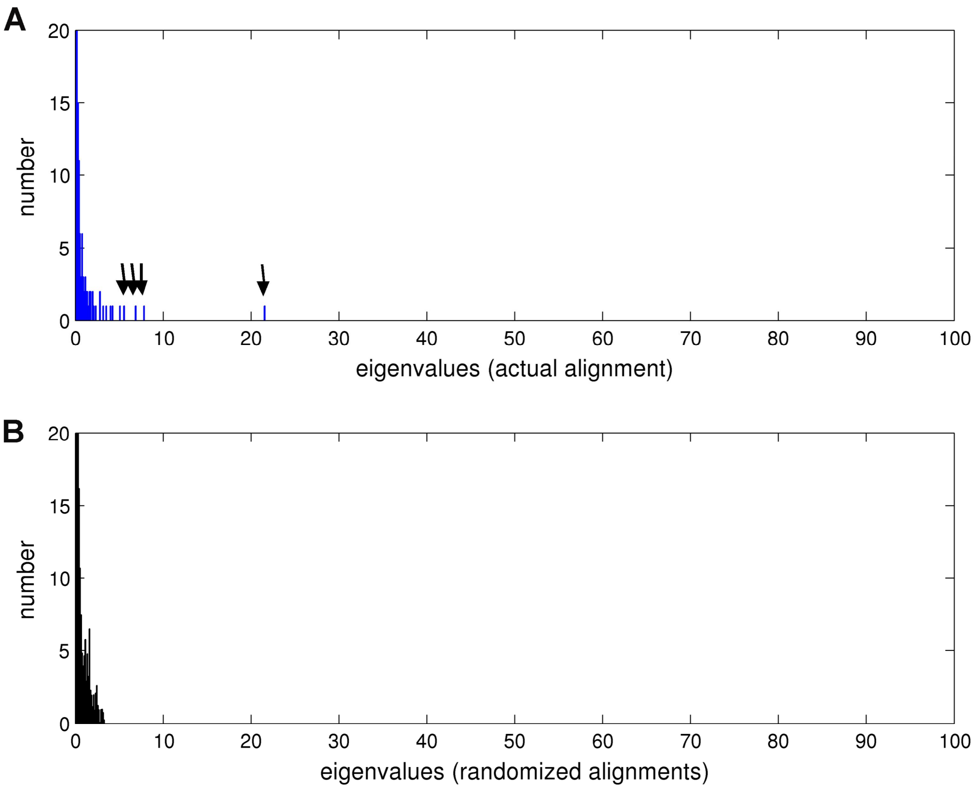

Figure S5: Histograms of eigenvalues for the actual alignment (A) and randomized alignment (B) of the type I glutaminase database are shown. Several eigenvalues may be considered statistically significant; however the top four values pointed out in (A) were used.
