# Supplementary material for: Importance of Hydrophobic Cavities in Allosteric Regulation of Formylglycinamide Synthetase: Insight from Xenon Trapping and Statistical Coupling Analysis
Source: PLoS One. 2013 Nov 1;8(11):e77781. doi: 10.1371/journal.pone.0077781 (PMC3815217; doi:10.1371/journal.pone.0077781)
Supplement: Figure S6 — Positions projected along the top four eigenvectors subsequent to independent component analysis step in Statistical Coupling Analysis of type I glutaminases. (PDF) [file pone.0077781.s006.pdf]

Figure S6

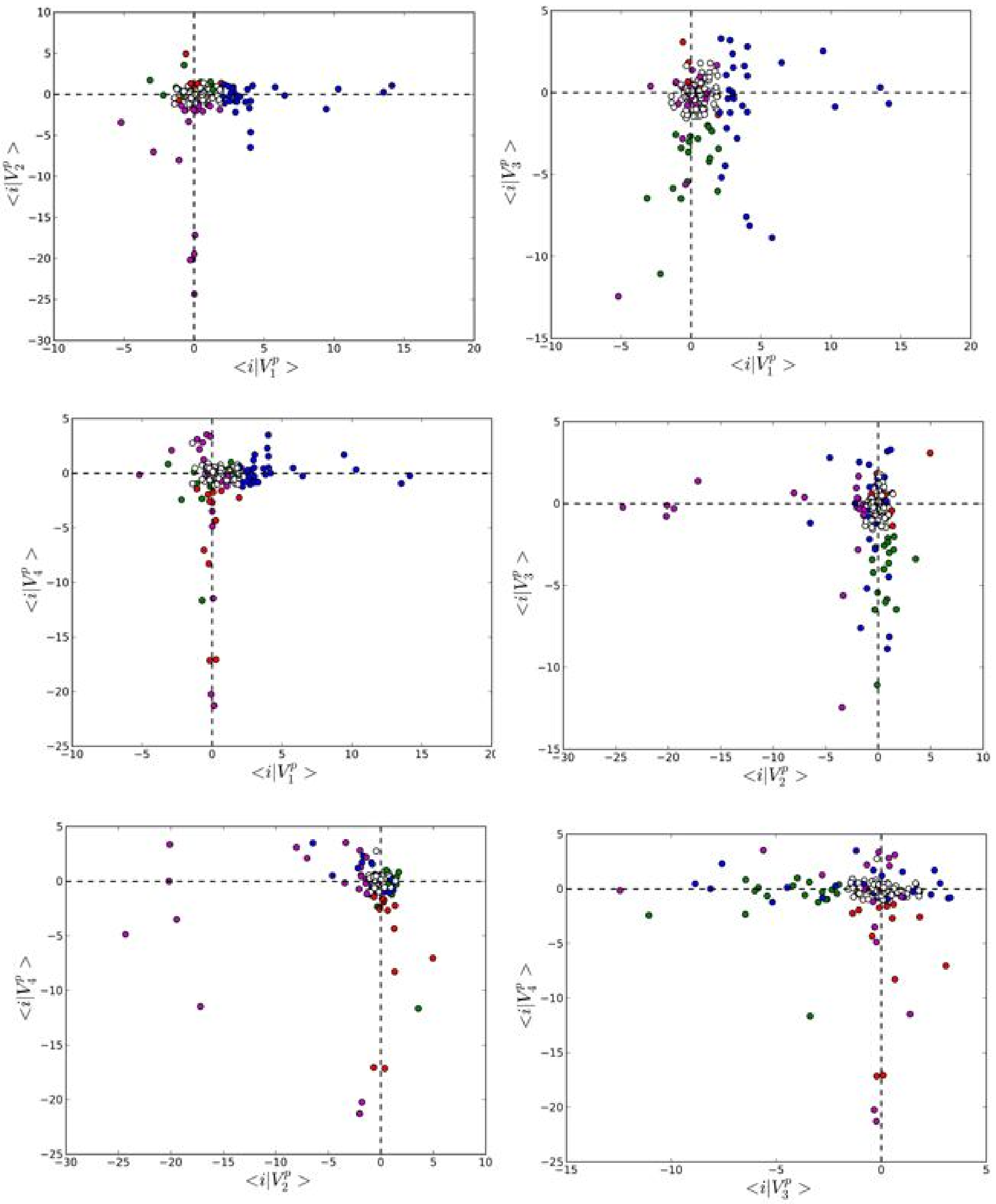

Figure S6: Positions projected along the top four eigenvectors subsequent to independent component analysis step in statistical coupling analysis of type I glutaminases. The magenta dots resolving along eigenvector 2 correspond to the magenta "allosteric" sector and the red dots resolving along eigenvector 4 correspond to the red "ammonia production" sector. No functional significance could be attributed to the blue and green sectors resolving along eigenvectors 1 and 3.
