# Supplementary figures and images for: Importance of Hydrophobic Cavities in Allosteric Regulation of Formylglycinamide Synthetase: Insight from Xenon Trapping and Statistical Coupling Analysis
Source: PLoS One. 2013 Nov 1;8(11):e77781. doi: 10.1371/journal.pone.0077781 (PMC3815217; doi:10.1371/journal.pone.0077781)

**Figure S7**

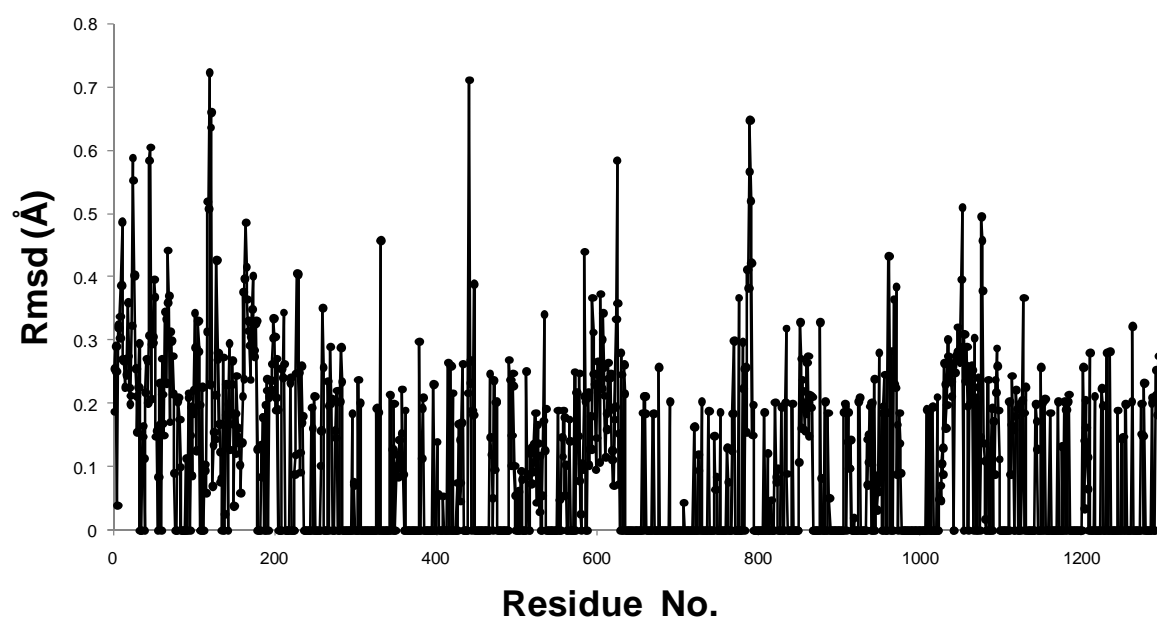

**Figure S7:** Per residue rmsd between StPurL (PDB code 1T3T) and StPurL-Xenon complex

Supplement: Figure S7 — Per residue RMSD between crystal structures of native StPurL and R1263A-Xenon complex. (PDF) [file pone.0077781.s007.pdf]
