# Supplementary material for: Importance of Hydrophobic Cavities in Allosteric Regulation of Formylglycinamide Synthetase: Insight from Xenon Trapping and Statistical Coupling Analysis
Source: PLoS One. 2013 Nov 1;8(11):e77781. doi: 10.1371/journal.pone.0077781 (PMC3815217; doi:10.1371/journal.pone.0077781)
Supplement: Figure S8 — Environment of the phenylalanine and tryptophan residues in the Xe1 cavity. (PDF) [file pone.0077781.s008.pdf]

Figure S8

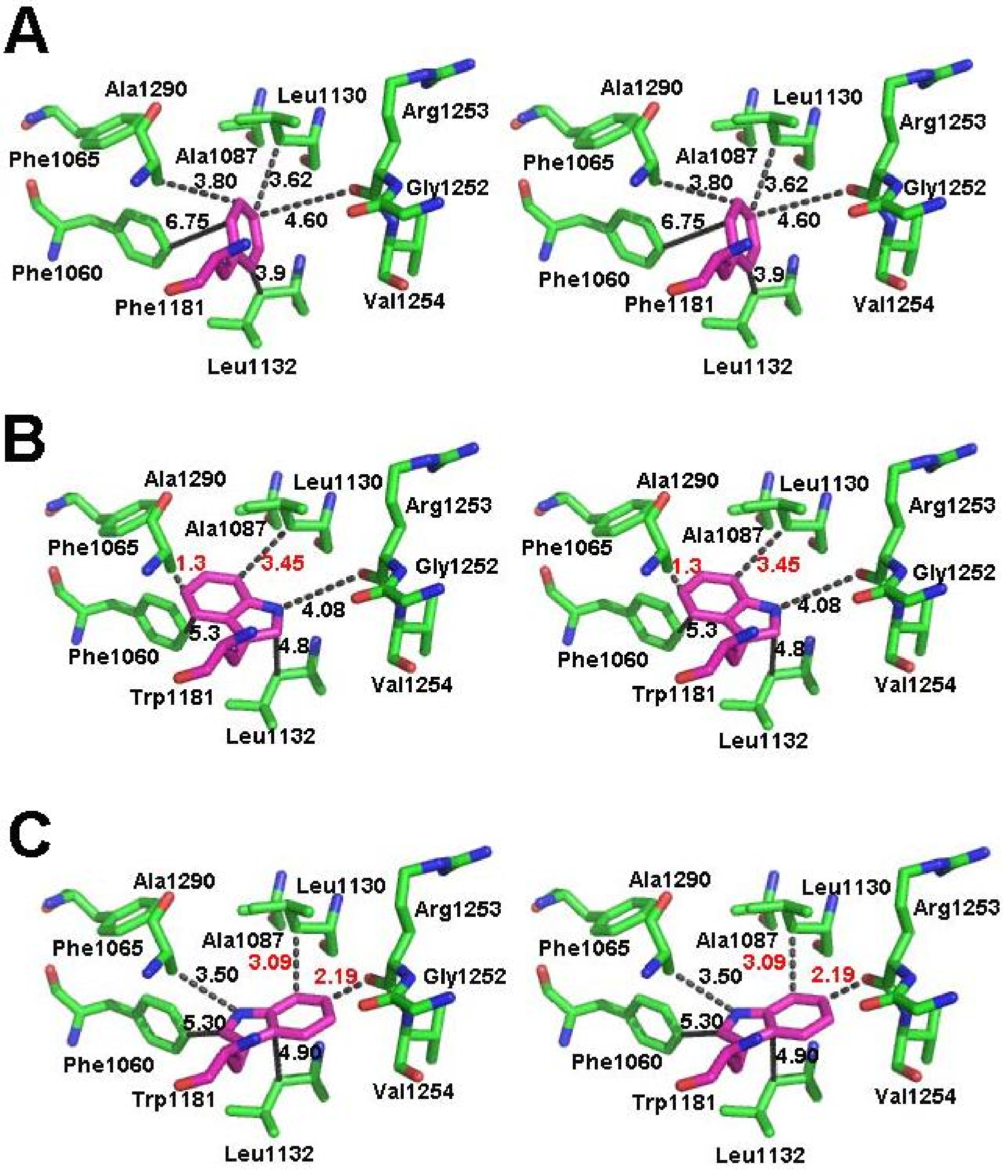

Figure S8: Stereoview of *insilico* mutations of phenylalanine and tryptophan residue in the cavity, mutations are shown with their carbon atoms as magenta sticks, closest distances of the mutated amino acid with the neighboring residues are depicted as dashed lines, other carbon atoms are labeled in green. (A) L118F mutant showing no steric clashes in the cavity. (B), (C) depict two alternate rotamers of tryptophan residue showing steric clashes with the neighboring pocket residue. The clash distances are labeled in red.
