# Supplementary material for: Importance of Hydrophobic Cavities in Allosteric Regulation of Formylglycinamide Synthetase: Insight from Xenon Trapping and Statistical Coupling Analysis
Source: PLoS One. 2013 Nov 1;8(11):e77781. doi: 10.1371/journal.pone.0077781 (PMC3815217; doi:10.1371/journal.pone.0077781)
Supplement: Figure S9 — Per residue RMSD between crystal structures of native StPurL and StPurL-F209W mutant. (PDF) [file pone.0077781.s009.pdf]

Figure S9

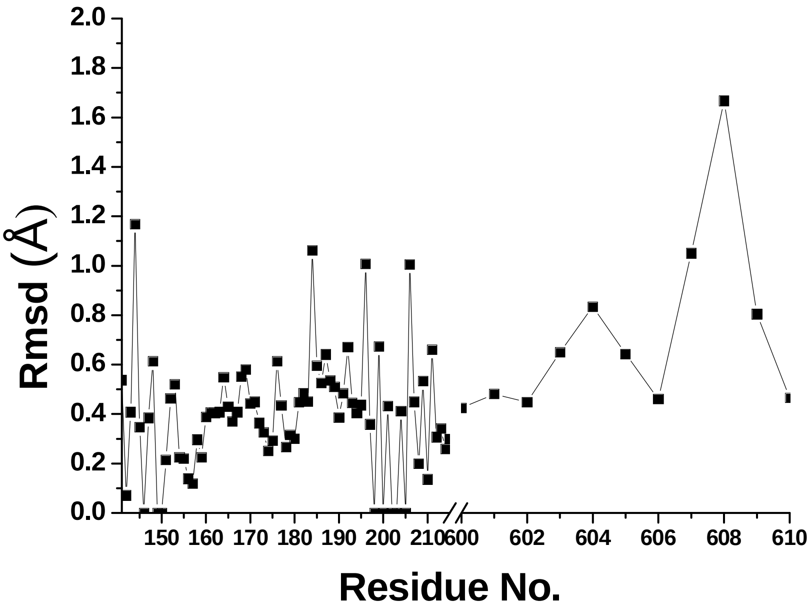

Figure S9: Per residue rmsd between StPurL (PDB code 1T3T) and StPurL-F209W mutant structure is depicted for residues near the xenon cavity 3 region.
