# Supplementary material for: Importance of Hydrophobic Cavities in Allosteric Regulation of Formylglycinamide Synthetase: Insight from Xenon Trapping and Statistical Coupling Analysis
Source: PLoS One. 2013 Nov 1;8(11):e77781. doi: 10.1371/journal.pone.0077781 (PMC3815217; doi:10.1371/journal.pone.0077781)
Supplement: Figure S10 — Green sector shown in surface representation with the xenon atoms highlighted. (PDF) [file pone.0077781.s010.pdf]

Figure S10

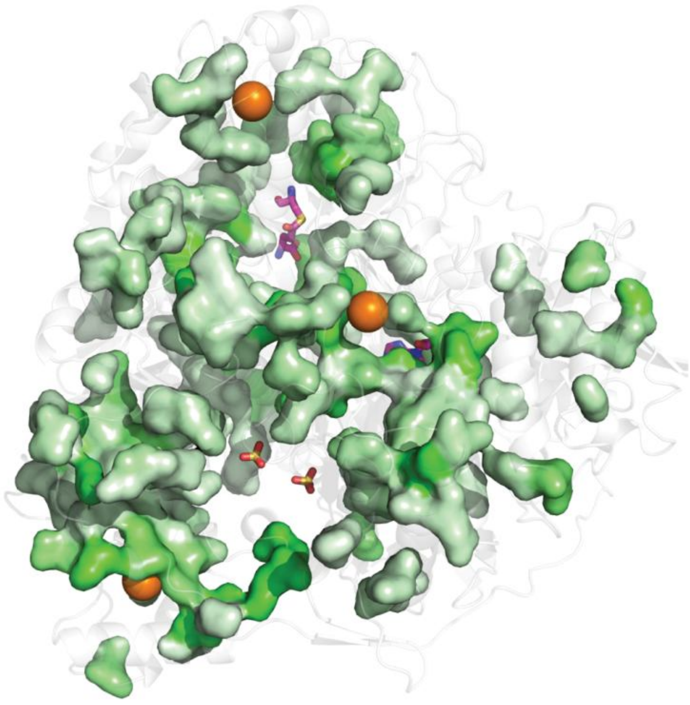

Figure S10: Sector residues of green sector are shown in surface representation with darker color representing higher SCA scores and lighter colors representing lower scores. Xe atoms are depicted as orange spheres. Locations of the two active sites and auxiliary ADP are shown in sticks.
