# Supplementary material for: Importance of Hydrophobic Cavities in Allosteric Regulation of Formylglycinamide Synthetase: Insight from Xenon Trapping and Statistical Coupling Analysis
Source: PLoS One. 2013 Nov 1;8(11):e77781. doi: 10.1371/journal.pone.0077781 (PMC3815217; doi:10.1371/journal.pone.0077781)
Supplement: Table S1 — Data processing and refinement statistics of StPurL-Xenon complex. (PDF) [file pone.0077781.s011.pdf]

Table S1

Table S1: Data processing and refinement statistics

**Data collection**	**StPurL-Xenon complex (PDB ID 4MGH)**
space group	$P6_5$
cell parameters (Å)	a =146.45, c = 141.68
resolution (Å)	2.65
multiplicity	21.7(21.0)
completeness (%)	99.3(97.5)
R_{sym} (%)	13.9(47.6)
I/σ	27.3(9.07)
Total no. of reflections	1091758
No. of unique reflections	50158
**Refinement**	
resolution range (Å)	19.99-2.65
total No. of reflections	48766
No. of reflections test set	1176
R_{work}/R_{free} (%)	15.3/20.2
No. of atoms	
total	10513
protein	9879
ligands	30
xenon	4
ion	82
water	518
**rmsd**	
bond lengths (Å)	0.02
bond angles (°)	1.78
Ramchandran Plot	
most favored region (%)	95.0
additionally allowed region (%)	4.4
outliers (%)	0.6

[a]values for the highest-resolution shell are given in parentheses
